# Chimeras of *Escherichia coli* and *Mycobacterium tuberculosis* Single-Stranded DNA Binding Proteins: Characterization and Function in *Escherichia coli*

**DOI:** 10.1371/journal.pone.0027216

**Published:** 2011-12-12

**Authors:** Sanjay Kumar Bharti, Kervin Rex, Pujari Sreedhar, Neeraja Krishnan, Umesh Varshney

**Affiliations:** 1 Department of Microbiology and Cell Biology, Indian Institute of Science, Bangalore, India; 2 Jawaharlal Nehru Centre for Advanced Scientific Research, Bangalore, India; University of Hyderabad, India

## Abstract

Single stranded DNA binding proteins (SSBs) are vital for the survival of organisms. Studies on SSBs from the prototype, *Escherichia coli* (*Eco*SSB) and, an important human pathogen, *Mycobacterium tuberculosis* (*Mtu*SSB) had shown that despite significant variations in their quaternary structures, the DNA binding and oligomerization properties of the two are similar. Here, we used the X-ray crystal structure data of the two SSBs to design a series of chimeric proteins (mβ1, mβ1′β2, mβ1–β5, mβ1–β6 and mβ4–β5) by transplanting β1, β1′β2, β1–β5, β1–β6 and β4–β5 regions, respectively of the N-terminal (DNA binding) domain of *Mtu*SSB for the corresponding sequences in *Eco*SSB. In addition, mβ1′β2_ESWR_ SSB was generated by mutating the *Mtu*SSB specific ‘PRIY’ sequence in the β2 strand of mβ1′β2 SSB to *Eco*SSB specific ‘ESWR’ sequence. Biochemical characterization revealed that except for mβ1 SSB, all chimeras and a control construct lacking the C-terminal domain (ΔC SSB) bound DNA in modes corresponding to limited and unlimited modes of binding. However, the DNA on *Mtu*SSB may follow a different path than the *Eco*SSB. Structural probing by protease digestion revealed that unlike other SSBs used, mβ1 SSB was also hypersensitive to chymotrypsin treatment. Further, to check for their biological activities, we developed a sensitive assay, and observed that mβ1–β6, *Mtu*SSB, mβ1′β2 and mβ1–β5 SSBs complemented *E. coli* Δ*ssb* in a dose dependent manner. Complementation by the mβ1–β5 SSB was poor. In contrast, mβ1′β2_ESWR_ SSB complemented *E. coli* as well as *Eco*SSB. The inefficiently functioning SSBs resulted in an elongated cell/filamentation phenotype of *E. coli*. Taken together, our observations suggest that specific interactions within the DNA binding domain of the homotetrameric SSBs are crucial for their biological function.

## Introduction

Single stranded DNA binding protein (SSB) plays a vital role in DNA replication repair and recombination [1]–[5]. SSBs are found in all organisms and, besides their crucial function in DNA transactions, they protect transiently generated single stranded DNA (ssDNA) from nuclease or chemical attacks [6]. Although the architecture of SSBs from different sources differs, they all possess an oligonucleotide binding fold (OB fold) in the N-terminal domain responsible for their oligomerization and ssDNA binding. Based on their oligomeric status, SSBs can be classified into monomeric, homo-dimeric, hetero-trimeric and homo-tetrameric proteins [6]–[12]. The C-terminal domain of the prokaryotic SSBs possesses a conserved acidic tail important in protein-protein interactions [13]–[16].

SSB from *Escherichia coli* (*Eco*SSB) has been an archetype to understand the biochemical, biophysical and the structural properties of the related SSBs [6]. *Eco*SSB consists of an N-terminal domain (∼115 amino acids) rich in β- sheets and a C-terminal domain without a defined tertiary structure [17], [18]. The C-terminal domain can be divided into a spacer region rich in glycine and proline residues, and a highly conserved region consisting of negatively charged residues (acidic tail). *Eco*SSB functions as a homo-tetramer consisting of four OB folds and interacts with ssDNA in different binding modes. In low salt (<20 mM NaCl) and high protein to DNA ratios, only two of the four subunits bind to ∼35 nucleotides in an unlimited cooperative manner to long ssDNA, known as SSB_35_ mode [19]–[22]. While in high salt (>0.2 M NaCl) and low protein to DNA ratios, all four subunits bind to ∼65 nucleotides in a limited cooperative manner to polynucleotides known as SSB_65_ mode [19]–[22]. The dynamic transition between these binding modes may be relevant for the *in vivo* function of SSBs [6], [23].

Unlike most other bacterial SSBs, SSBs from *Deinococcus/Thermus* group have been characterized to form homodimers [24]–[28]. However, in these SSBs, each monomer contains two OB folds. Studies with *Deinococcus radiodurans* SSB (*Dra*SSB) show that the mechanism of DNA wrapping onto it is not identical to that of *Eco*SSB [27]. However, the DNA binding affinity, rate constant and association mechanisms of *Dra*SSB are similar to those of *Eco*SSB. Interestingly, *Dra*SSB complements *E. coli* for the essential function of SSB [26]. SSB from *Helicobacter pylori* which is closer to *Eco*SSB for its various properties is also known to function in *E. coli* [29], [30].

SSB from *M. tuberculosis* (*Mtu*SSB) shares ∼30% identity and ∼39% similarity with *Eco*SSB in its primary sequence. Although the dynamics and the mode of DNA binding to *Mtu*SSB have not been studied in detail, the initial biochemical characterization has shown that like *Eco*SSB, *Mtu*SSB is a homotetramer and binds to ssDNA in two modes similar to *Eco*SSB [31]. The three-dimensional structure of SSB from *M. tuberculosis* suggested significant variability in its quaternary structure. The *Mtu*SSB has unique dimeric interface facilitated by the clamp structures formed by β6 strands of the interacting subunits [32]. Such structural differences were also observed in SSBs of other mycobacteria (*M. smegmatis* and *M. leprae*) [33], [34].

To further our understanding of the structure-function relationship of eubacterial SSBs, in this study, we have generated a number of chimeric SSBs by swapping different regions of *Eco*SSB and *Mtu*SSB and analyzed them for their *in vitro* and, *in vivo* properties using a sensitive assay system designed in this study.

## Results

### Generation of chimeric SSBs

**Figure 1:**
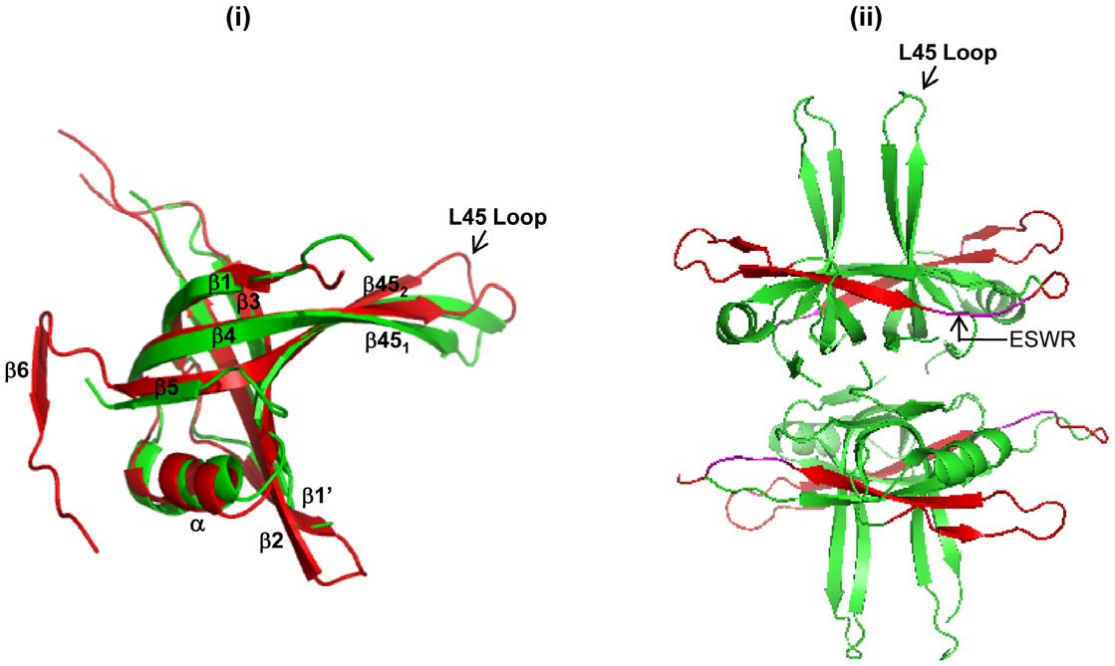
Comparison of tertiary and quaternary structures of *Eco*SSB and *Mtu*SSB. (i) Tertiary structures of *Eco*SSB and *Mtu*SSB are represented in green and red, respectively. The secondary structure elements including the β6, which facilitates formation of a hook like structure in *Mtu*SSB [32] are as shown. (ii) Quaternary structure of *Eco*SSB highlighting the regions away from the subunit-subunit interface. A ribbon diagram of SSB (PDB 1KAW) is depicted through PyMol (http://www.pymol.org/). The region corresponding to β1′ and β2 is shown in red.

**Figure 2:**
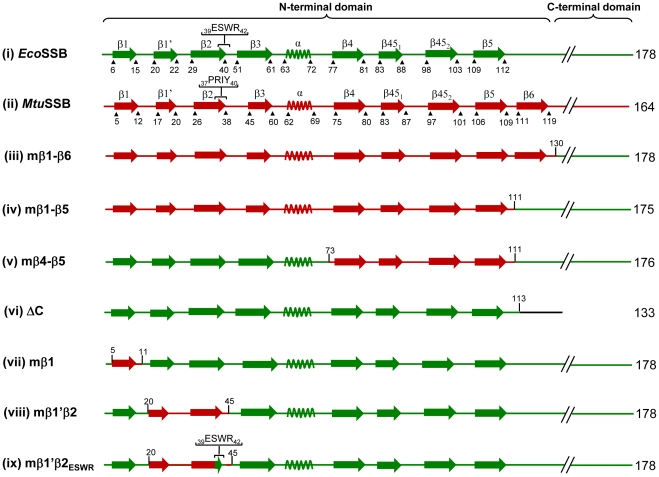
Schematic representation of the secondary structure elements. *Mtu*SSB and *Eco*SSB are represented in red and green colors, respectively. The beginning and end of each structural unit are numbered. The C-terminal domains are shown by discontinuous lines. In the chimeric proteins, various structural elements (N-terminal domain) are indicated in the respective colors.

Chimeric constructs were designed based on the three dimensional structures of *Eco*SSB and *Mtu*SSB (Figure 1). Details of generation of the chimeric constructs are provided in the supporting material (Methods S1), and shown schematically is Figure 2. In our earlier study [35], we generated a chimeric *MtuEco*SSB which has been renamed as mβ1–β6 SSB (Figure 2, iii), possessing the N-terminal region (amino acids 1–130; the initiating methionine is numbered as 1) from *Mtu*SSB and the C-terminal region (131–178) from *Eco*SSB. The crystal structure of *Mtu*SSB [32] revealed a novel hook like structure formed by the presence of the β6 strand, an element absent from *Eco*SSB (Figure 1, i; Figure 2, i and ii). Hence, the mβ1–β5 SSB containing β1 to β5 strands (first 111 amino acids) from *Mtu*SSB and the remainder of the sequence from *Eco*SSB (Figure 2, iv) was also designed.

In other constructs, various secondary structural elements in the N-terminal domain of *Eco*SSB were replaced with the corresponding regions of *Mtu*SSB (Figure 2). The mβ4–β5 SSB, contained the first 73 amino acids comprising β1, β2, β3 strands and the α-helix from *Eco*SSB, and amino acids 74 to 111 comprising β4, β45_1_, β45_2_ and β5 strands from *Mtu*SSB, followed by amino acids 112 to the end of the protein from *Eco*SSB (Figure 2, v). In the mβ1 SSB (Figure 2, vii) amino acids 6–11in the β1 strand of *Eco*SSB were substituted with the corresponding *Mtu*SSB sequence (Table 1, S3).

**Table 1:** List of strains, plasmids and DNA oligomers.

Strain/plasmids/DNA oligomer	Details	Reference
***E. coli*** ** strains**		
RDP 317	*E. coli* (Δ*ssb*::*kan*) harboring pRPZ150 (ColE1 ori, Tet^R^)	[41], [42]
RDP 317-1	*E. coli* (Δ*ssb*::*kan*) harboring pHYD*Eco*SSB (ColE1 ori, Cam^R^) whose replication is dependent upon the presence of IPTG.	This work
TG1	An *E. coli* K strain, F^-^ LAM^-^ *rph*-1	[49]
BL21 (DE3)	Harbors T7 RNA polymerase gene under the control of LacI	Novagen
**Plasmids**		
pTrc*Eco*SSB	pTrc99C containing *Eco-ssb* ORF	[35]
pTrc*Eco*SSB (G114A)	pTrc99C containing *Eco-ssb* ORF wherein G114A mutation was incorporated to generate NheI site. This mutant was functional in the plasmid bumping assay.	This work
pTrc*Mtu*SSB	pTrc99C containing *Mtu-ssb* ORF	[35]
pTrc*Mtu*SSB(R111A)	pTrc99C containing *Mtu-ssb* ORF wherein R111A mutation was generated to create NheI site.	This work
pHYD*Eco*SSB	Derived from pHYD1621 containing IPTG dependent ColE1 ori of replication (a gift from Dr. J. Gowrishanker, CDFD, Hyderabad India). EcoRV to PstI fragment from pTrc*Eco*SSB was subcloned cloned into Ecl136II and PstI digested pHYD1621.	This work
pTrc mβ1–β6 SSB	pTrc99C containing chimeric SSB, wherein the first 130 amino acids are from *Mtu*SSB and the remaining (131 to 178) are from *Eco*SSB (renamed from *MtuEco*SSB)	[35]
pTrc mβ1–β5 SSB	pTrc99C containing chimeric SSB wherein the first 111 amino acids are from *Mtu*SSB(R111A) and the remaining (112 to 176) are from *Eco*SSB.	This work
pTrcΔC SSB	pTrc99C containing chimeric SSB wherein the first 113 amino acids are from *Eco*SSB, and the remaining (114 to 133) are due to *Mtu*SSB or vector encoded amino acids.	This work
pTrc mβ4–β5 SSB	pTrc99C containing chimeric SSB wherein the first 73 amino acids are from *Eco*SSB (containing R73A mutation) and the remainder (74 to 176) from mβ1–β5 SSB.	This work
pTrc mβ1 SSB	pTrc99C containing chimeric SSB wherein the first 5 amino acids are from *Eco*SSB, amino acids 6 to 11 are from *Mtu*SSB (corresponding to residues 4–9 in *Mtu*SSB)) and the remaining (12 to 178) are from *Eco*SSB.	This work
pTrc mβ1′β2 SSB	pTrc99C containing chimeric SSB wherein the first 20 amino acids are from *Eco*SSB, amino acids 21 to 45 (corresponding to residues 19–43 in *Mtu*SSB) are from *Mtu*SSB and the remaining (46 to 178) are from *Eco*SSB.	This work
pTrc mβ1′β2_ESWR_SSB	pTrc99C containing mβ1′β2 SSB wherein the _39_PRIY_42_ (corresponding to residues 37–40 in *Mtu*SSB) of mβ1′β2 SSB was changed with *Eco*SSB specific sequence _39_ESWR_42_.	This work
pET11D	pET11D (ColE1 ori, Amp^R^). A T7 RNA polymerase based expression vector.	Novagen
pET mβ1–β6 SSB	pET11D containing mβ1–β6 SSB	This work
pET mβ1–β5 SSB	pET11D containing mβ1–β5 SSB	This work
pET mβ4–β5 SSB	pET11D containing mβ4–β5 SSB	This work
pET mβ1 SSB	pET11D containing mβ1 SSB	This work
pET ΔC SSB	pET11D containing ΔC SSB	This work
pUC mβ1 SSB	Eco32I-HindIII fragment from pTrc mβ1 SSB was mobilized to Ecl136II and HindIII digested pUC 18R (Amp^R^, multicopy plasmid).	This work
pBAD/His B	pBAD/HisB plasmid (ColE1 ori, Amp^R^). An expression vector containing arabinose inducible promoter.	Invitrogen
pBAD ΔC SSB	pBAD/HisB containing ΔC SSB	This work
pBAD mβ4–β5SSB	pBAD/HisB containing mβ4–β5 SSB	This work
pBAD mβ1 SSB	pBAD/HisB containing mβ1 SSB	This work
pBAD mβ1′β2 SSB	pBAD/HisB containing β1′β2 SSB	This work
pBAD mβ1′β2_ESWR_ SSB	pBAD/HisB containing β1′β2_ESWR_ SSB	This work
**DNA oligomer**		
79 mer ssDNA	5′gcactagtgcggatagccccgtgttgttgtctgacccccgaccccgacggcaatgcggggcaatcccctggaggcctgc 3′	This work

The above constructs possessed substitutions of *Eco*SSB regions involved in subunit-subunit interactions. Hence, we generated mβ1′β2 SSB wherein amino acids 21 to 45 comprising β1′ and β2 strands positioned in the exterior of the tetramer (Figure 1, ii), were exchanged with the corresponding sequences from *Mtu*SSB (Figure 2, viii). The mβ1′β2_ESWR_ SSB was generated from mβ1′β2 SSB by replacing four amino acids of the *Mtu*SSB origin at positions 39 to 42 (PRIY, in the β2 strand) with the *Eco*SSB specific sequence, ESWR (Figure 2, ix). And, a clone with deletion of C-terminal domain of *Eco*SSB, ΔC SSB (Figure 2, vi), was identified serendipitously during sequence analysis of the generated constructs.

### Oligomerization status of chimeric SSBs

**Figure 3:**
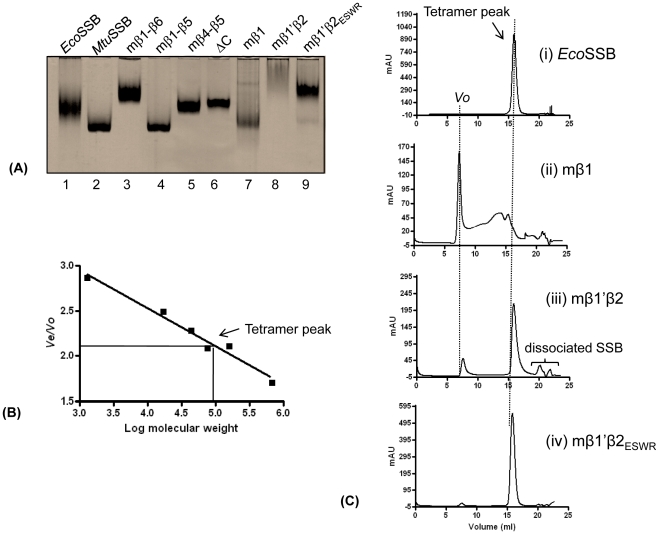
Oligomeric status of SSB proteins. (**A**) Analysis of SSBs (∼2 µg each) on native PAGE (12%). Lanes: 1, *Eco*SSB; 2, *Mtu*SSB; 3, mβ1–β6 SSB; 4, mβ1–β5 SSB; 5, mβ4–β5 SSB; 6, ΔC SSB; 7, mβ1 SSB; 8, mβ1′β2 SSB; and 9, mβ1'β2_ESWR_ SSB. (**B**) Standard curve *Ve*/*Vo versus* log molecular size markers. *Ve* corresponds to the peak elution volume of proteins and *Vo* represents the void volume of the column determined using blue dextran (2,000 kDa). The protein size markers (Materials and Methods) were used to make the plot. The tetramer peak corresponding to *Eco*SSB is indicated. (**C**) The gel filtration chromatography elution profiles of, (i) *Eco*SSB; (ii) mβ1 SSB; (iii) mβ1′β2 SSB and (iv) mβ1′β2_ESWR_ SSB are shown. Tetramer peak and *Vo* are indicated by dashed vertical lines.

Analysis of the purified SSBs using native PAGE is shown in Figure 3A. On such a gel, *Eco*SSB and *Mtu*SSB were shown to migrate as homotetramers [31], [35]. The migration of many chimeras was comparable to *Eco*SSB or *Mtu*SSB suggesting their homotetrameric nature. However, we observed diffuse migration of mβ1 and mβ1′β2 SSBs, suggesting alteration(s) in their oligomerization/folding properties. Interestingly, introduction of *Eco*SSB specific ‘ESWR’ sequence in mβ1′β2_ESWR_ SSB in place of *Mtu*SSB specific ‘PRIY’ sequence (in mβ1′β2 SSB), restored its mobility as a tetramer (Figure 3A, lanes 8 and 9). To further analyze the oligomerization status of the chimeric SSBs, we performed gel filtration chromatography wherein *Eco*SSB eluted as tetramer (Figure 3C, i). The oligomeric nature of other SSBs was determined from a standard plot of *Ve*/*Vo versus* log molecular weight (Figure 3B). Consistent with the diffuse mobility of mβ1 SSB in native PAGE (Figure 3A), it eluted in the void volume suggesting alteration in its oligomerization/folding properties (Figure 3C, ii). On the other hand, while a fraction of the mβ1′β2 SSB eluted as tetramer, its elution continued beyond the tetramer peak suggesting poor tetramerization (Figure 3C, iii). However, as expected from the native gel analysis, introduction of ESWR sequence in mβ1′β2 SSB (in place of PRIY sequence) restored its oligomeric status as a tetramer, and it eluted same as *Eco*SSB (Figure 3C, iv). Other chimeras eluted as tetramers (Figure S1). As expected, the elution profile of ΔC SSB suggested it to be tetramer but smaller in molecular weight.

### DNA binding activity of chimeric SSBs

**Figure 4:**
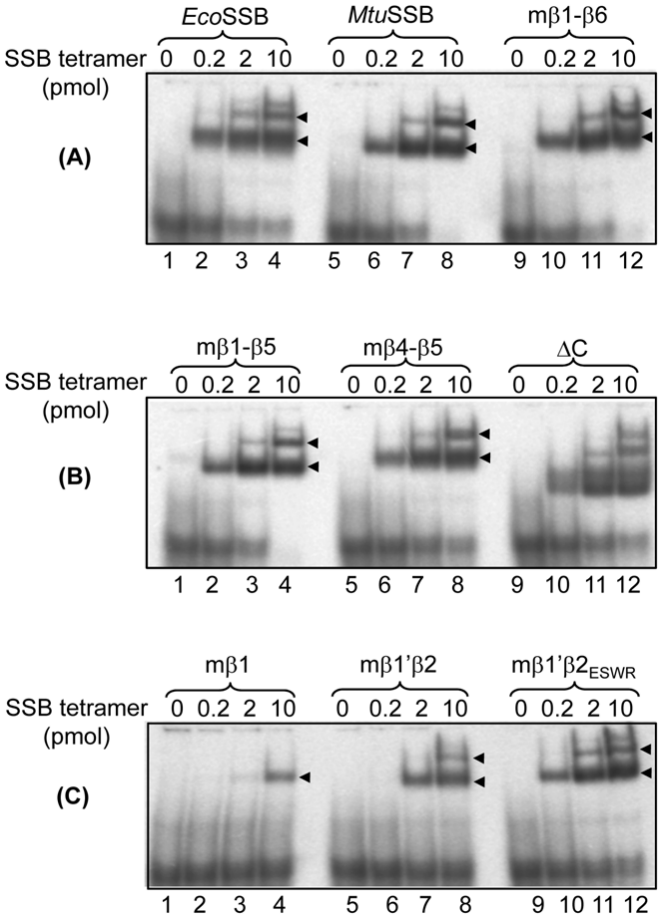
Electrophoretic mobility shift assays using ^32^P labeled 79mer ssDNA. DNA (1 pmol) was mixed with 0.2 pmol (lanes 2, 6 and 10); 2 pmol (lanes 3,7 and 11) and 10 pmol (lanes 4, 8 and 12) of SSBs (as indicated) for 30 min and analyzed on native PAGE (8%).

Using electrophoretic mobility shift assays (EMSA) [31], [35] we observed that all SSBs formed protein-DNA complexes (Figures 4A–C). However, the complex formation with mβ1 SSB was extremely poor and detectable only at the highest concentration of the protein (Figure 4C, lanes 1–4). Also, in such assays, *Eco*SSB and *Mtu*SSB have been shown to bind longer DNA oligomers in two forms corresponding to SSB_35_ and SSB_56/65_ binding modes of *Eco*SSB [31]. As a 79mer DNA was used, at higher molar ratios of SSB to DNA, two major complexes were seen with all except mβ1 SSB which showed poor binding, and the ΔC SSB showed additional complexes (compare lanes 3 and 4, with 2; 7 and 8 with 6; 11 and 12 with 10 in Figures 4A–C, respectively). However, at low SSB to DNA ratios, a single complex of mobility corresponding to SSB_56/65_ was seen (compare lanes 2 with 1; 6 with 5; and 10 with 9 in Figures 4A and 4B). Highly compromised binding of mβ1 SSB (Figure 4C, lanes 1–4) is consistent with its altered oligomerization/folding properties (Figure 3). However, the DNA binding ability of mβ1′β2 SSB (Figure 4C, lanes 5 to 8) which showed weak tetramerization (Figure 3C) appeared not as compromised. The nature of the complexes seen with ΔC SSB, was not investigated. However, the presence of multiple bands (Figure 4B, lane 12) may indicate that the C-terminal domain may contribute to remodeling DNA binding predominantly in SSB_35_ and SSB_56/65_ modes.

### Susceptibility of chimeric SSBs to chymotrypsin digestion

**Figure 5:**
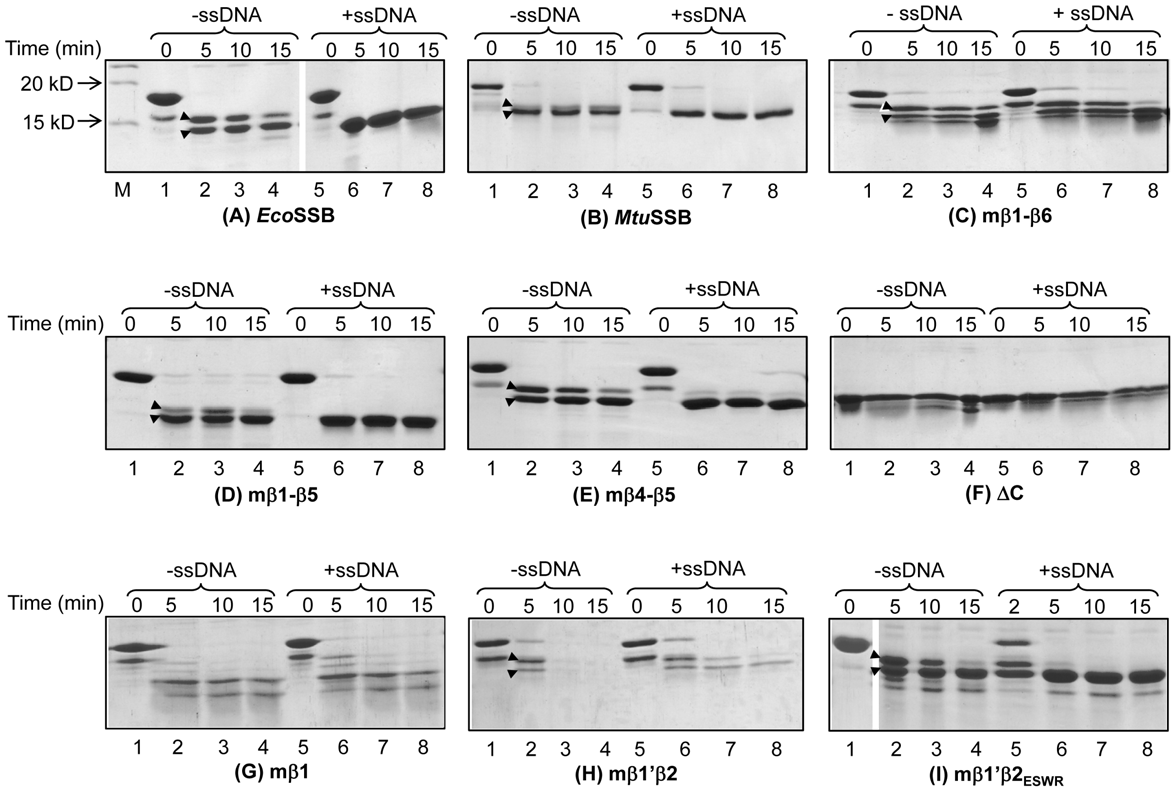
Digestion of SSBs with chymotrypsin. Approximately 2 µg of SSBs (as shown), in the absence or presence of the sheared and denatured genomic DNA were incubated with chymotrypsin for the indicated times and analyzed on SDS-PAGE (17.5%). M represents molecular size markers.

Chymotrypsin which cleaves at the carboxyl side of the aromatic amino acids (F, Y and W) has 14 cleavage sites in *Eco*SSB. While the sites within the N-terminal domain are protected by a well formed structure, the sites within the C-terminal domain are sensitive to cleavage. As shown earlier [18], the sites at 136 and 148 positions are particularly prone to cleavage, and at early time points in the reaction, two bands corresponding to ∼14 kD and ∼15 kD are seen. Upon DNA binding the conformational changes in the C- terminal domain make the site at position 136 more accessible and a single product corresponding to ∼14 kD is seen [18]. We used this assay to probe for conformational changes in the chimeric SSBs upon ssDNA binding. Chymotrypsin cleavage pattern of the free and DNA bound *Eco*SSB (Figure 5A) was the same as reported [18]. The digestion of *Mtu*SSB also resulted in two products migrating as a doublet; and the presence of ssDNA resulted in a single band corresponding to the lower band of the doublet (Figure 5B). The cleavage patterns of the chimeric SSBs followed the same trend, resulting in relative accumulation of the smaller product (bands marked with arrowheads) upon DNA binding (Figures 5C–E and I). The ΔC SSB lacking C terminal domain, as expected, did not result in change in the digestion pattern (Figure 5F). The mβ1 SSB, defective in oligomerization/folding, was more sensitive to digestion. And, consistent with its poor DNA binding (Figure 4C) it did not show a relative accumulation of the smaller sized product upon DNA binding (Figure 5G). While mβ1′β2 SSB (Figure 5H) was somewhat more sensitive to chymotrypsin than the other constructs (Figures 5A–E), substitution of the PRIY sequence in its β2 strand with ESWR (in mβ1′β2_ESWR_ SSB) rescued it from its protease sensitivity (Figure 5I), as it did its oligomerization and DNA binding properties (Figure 3A, lanes 8 and 9; 3C, iii and iv; and Figure 4C, lanes 5–12). These observations lend further support to the observations (Figures 3 and 4) that mβ1 and mβ1′β2 SSBs suffer from structural alterations.

### Fluorescence titration of SSBs

Fluorescence reverse titrations have been used to determine the binding site sizes of SSBs [19]–[22], [27]. We performed such experiments to determine the kinetic parameters for DNA binding to various SSBs using poly (dT) in 50 and 200 mM NaCl. Except for mβ1 SSB which revealed altered structure, highly compromised DNA binding, and high sensitivity to chymotrypsin (Figures 3, 4, 5), other SSBs resulted in similar fluorescence quenching (Figure S2). We then processed [36]–[40] these data to estimate values of maximal fluorescence quenching (*Q*_max_), binding site size (*n*), binding constant (*K*_obs_) and co-operativity (*ω*) for each of the chimeras except for mβ1 SSB (Table 2). The values of *Q*_max_, *n* and *ω* of *Eco*SSB, *Mtu*SSB and the other SSBs were comparable. As reported previously [19], we observed that the binding site size of *Eco*SSB increased from 50 to 68 when NaCl concentration was changed from 50 mM to 200 mM, respectively. On the contrary, *Mtu*SSB did not show a similar increase in binding site size upon increase in salt concentration. Binding site size of *Mtu*SSB was observed to be 72 in 50 mM NaCl and it changed to 76 in 200 mM NaCl. The chimeric SSBs (except mβ1′β2_ESWR_ SSB) exhibited comparable binding site sizes in the presence of 200 mM NaCl. At 50 mM salt, ssDNA binding with mβ1′β2, mβ1′β2_ESWR_, mβ1–β6 SSBs showed binding site sizes comparable to *Eco*SSB. The binding site sizes of mβ1–β5, mβ4–β5 and ΔC SSBs were 30, 32 and 36, respectively in 50 mM salt. DNA binding experiments in the presence of 50 mM and 200 mM NaCl suggested that *Mtu*SSB may follow a different DNA binding path than the *Eco*SSB.

**Table 2:** Kinetic parameters of SSB interaction with poly dT.

SSB constructs	Buffer A containing ∼200 mM NaCl				Buffer A containing ∼50 mM NaCl			
	*Q*_max_	*n*	*K*_obs_	ω	*Q*_max_	*n*	*K*_obs_	Ω
*Eco*SSB	88.21	68.0	0.007	0.53	78.1	50	0.0013	0.55
*Mtu*SSB	77.90	76.0	0.055	0.51	62.25	72	0.0152	0.53
mβ1′–β2 SSB	83.80	76.0	0.039	0.51	68.94	52	0.0013	0.55
mβ1′–β2_ESWR_	85.39	64.0	0.004	0.53	71.92	46	0.0001	0.60
mβ1–β6 SSB	75.69	74.0	0.029	0.52	68.83	52	0.0024	0.54
mβ1–β5 SSB	81.19	72.0	0.019	0.52	80.27	30	0.0004	0.60
mβ4–β5 SSB	80.08	78.0	0.057	0.51	69.93	32	0.0038	0.60
ΔC SSB	93.40	74.0	0.02	0.52	69.58	36	0.0004	0.58
mβ1 SSB	-ND-	-ND-	-ND-	-ND-	-ND-	-ND-	-ND-	-ND-

Estimated binding constant (*K*_obs_ mM^−1^), maximal fluorescence quenching (*Q*_max_), binding site size (*n*) and co-operativity (*ω*) are as shown. ND: Not determined.

### *In vivo* complementation analysis of the chimeric SSBs

To further characterize the chimeric SSBs, it was of interest to determine if they complemented *E. coli* for the *in vivo* function of SSB. We first used the ‘plasmid bumping’ method [35], [[41]; and Methods S2] where the test *ssb* construct (in a ColE1 ori plasmid, Amp^R^) was introduced in a *Δssb* (*ssb*::*kan*) strain (RDP317, Kan^R^) of *E. coli* [42] harboring a wild-type *ssb* gene on another ColE1 ori plasmid (pRPZ150, Tet^R^), and the transformants cultured for multiple rounds in the presence of Amp and Kan. As both plasmids possess ColE1 ori, under the growth conditions, the strain would lose the original Tet^R^ plasmid if the test *ssb* plasmid (Amp^R^) substituted for the essential function of *Eco*SSB [35], [41] giving rise to Amp^R^Tet^S^ population. Using this assay, we observed that besides the positive control of *Eco*SSB, only mβ1′β2_ESWR_ SSB resulted in ‘bumping’ of the original plasmid (Table S1). However, it may be that in this assay a weakly complementing SSB construct does not ‘bump’ the original plasmid due to fitness disadvantage. Hence, a new assay wherein pHYD*Eco*SSB (Cam^R^) construct sustained the *E coli* RDP317-1 strain (Kan^R^) was developed. As the replication of pHYD*Eco*SSB is dependent on the presence of IPTG, its withdrawal from the growth medium results in the loss of the plasmid and failure of the strain growth unless sustained by the test SSB.

**Figure 6:**
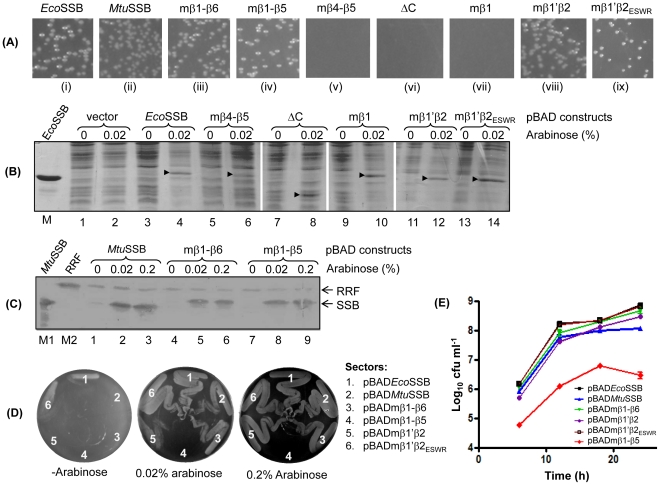
Functional analysis of SSBs. (**A**) Transformants of chimeric SSB constructs in *E. coli* RDP 317-1 in the presence of 0.02% arabinose (and absence of IPTG). Panels: (i) pBAD*Eco*SSB, (ii) pBAD*Mtu*SSB, (iii) pBADmβ1–β6, (iv) pBADmβ1–β5, (v) pBADmβ4–β5, (vi) pBADΔC, (vii) pBADmβ1, (viii) pBADmβ1′β2, and (ix) pBADmβ1′β2_ESWR_. (**B**) Expression analysis of SSBs in *E. coli* TG1. SDS-PAGE analysis of 10 µg total cell proteins of the transformants harboring SSB constructs as indicated on top of the gel. (**C**) Immunoblot analysis of 10 µg total cell proteins of transformants as indicated on top of the blot using antibodies against *Mtu*SSB and *Eco*RRF (host protein used as loading control). Lane M, is the marker lane containing 80 ng and 180 ng of *Mtu*SSB and *Eco*RRF, respectively. (**D**) Streaking of the overnight cultures of the various transformants obtained in panel (**A**) on LB-agar containing Kan, Amp and arabinose (0.0–0.2%) and incubated at 37°C for ∼12 h. Sectors: 1, pBAD*Eco*SSB; 2, pBAD*Mtu*SSB; 3, pBADmβ1–β6; 4, pBADmβ1–β5; 5, pBADmβ1′β2; and 6, pBADmβ1′β2_ESWR_ (**E**) Cell viability of RDP 371-1 supported with various SSB constructs. Colony forming units (cfu) were determined at 6, 12, 18 and 24 h of the culture growth.

The SSB constructs were subcloned into a ColE1 ori (Amp^R^) plasmid wherein their expression was inducible by arabinose (the pBAD series of constructs, Table 1) and introduced into the RDP317-1 strain (Kan^R^) harboring pHYD*Eco*SSB (Cam^R^). Transformants were selected on LB agar containing Kan, Amp and 0.02% arabinose (Figure 6A). Six of the constructs (*Eco*SSB, *Mtu*SSB, and the mβ1–β6, mβ1–β5, mβ1′β2 and mβ1′β2_ESWR_ SSBs) yielded transformants, indicating that they functioned in *E. coli*. The remaining three constructs (ΔC, mβ1 and mβ4–β5 SSBs) did not yield any transformants. When checked for their expression, all chimeric SSBs showed expression in *E. coli* TG1 (Figures 6B and 6C). The transformants for ΔC, mβ1 and mβ4–β5 SSBs constructs could be obtained in the presence of IPTG (to allow replication of pHYD*Eco*SSB). Unfortunately, these transformants (unlike the ones obtained with *Eco*SSB, *Mtu*SSB, and mβ1–β5, mβ1–β6, mβ1′β2, mβ1′β2_ESWR_ SSB constructs) retained the support plasmid (Cam^R^) even after their subsequent growth in the absence of IPTG. The reasons for this observation are unclear.

**Figure 7:**
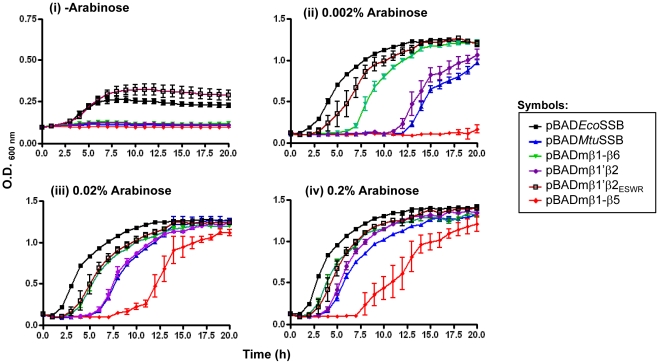
Growth of *E. coli* RDP317 (Δ*ssb::kan*) supported by various SSBs in the absence (panel i) or presence of 0.002, 0.02 or 0.2% arabinose (panels ii–iv, respectively). Averages (±SEM) of the growth of three independent colonies are plotted.

When the transformants obtained with *Eco*SSB, *Mtu*SSB, and mβ1–β5, mβ1–β6, mβ1′β2, and mβ1′β2_ESWR_ SSB constructs, were streaked on a fresh plate, except for the mβ1–β5 construct, the other constructs supported efficient growth upon induction of SSB expression by arabinose (Figure 6D). Interestingly, when grown in liquid medium, even the transformant harboring mβ1–β5 SSB reached saturation. However, total viable count determination revealed that viability of cells harboring mβ1–β5 SSB was severely compromised (Figure 6E). The growth curve experiment (Figure 7) revealed that the mβ1′β2_ESWR_ SSB supported *E. coli* growth nearly as well as *Eco*SSB (Figure 7, panel ii). The growth in the presence of mβ1′β2 SSB and *Mtu*SSB was weak. Importantly, mβ1–β6 SSB wherein the C-terminal domain of *Mtu*SSB was replaced with that from *Eco*SSB, supported better growth (Figure 7, panel ii). As *Mtu*SSB and mβ1–β6 SSB are expressed to similar levels (Figure 6C), this observation is consistent with the importance of interactions of the various cellular proteins with the C-terminal of the homologous *Eco*SSB [13]–[16]. In the absence of induction of SSB expression, the basal level expression of only the wild-type *Eco*SSB and the mβ1′β2_ESWR_ SSB resulted in some visible growth (Figure 7, panel i). Furthermore, we observed that with the increase in the concentration of the inducer, the lag phases in the cases of less efficiently functioning SSBs (*Mtu*SSB, mβ1–β6, mβ1′β2 and mβ1–β5) decreased suggesting a dose dependent complementation of an *E. coli* Δ*ssb* strain by these SSBs.

**Figure 8:**
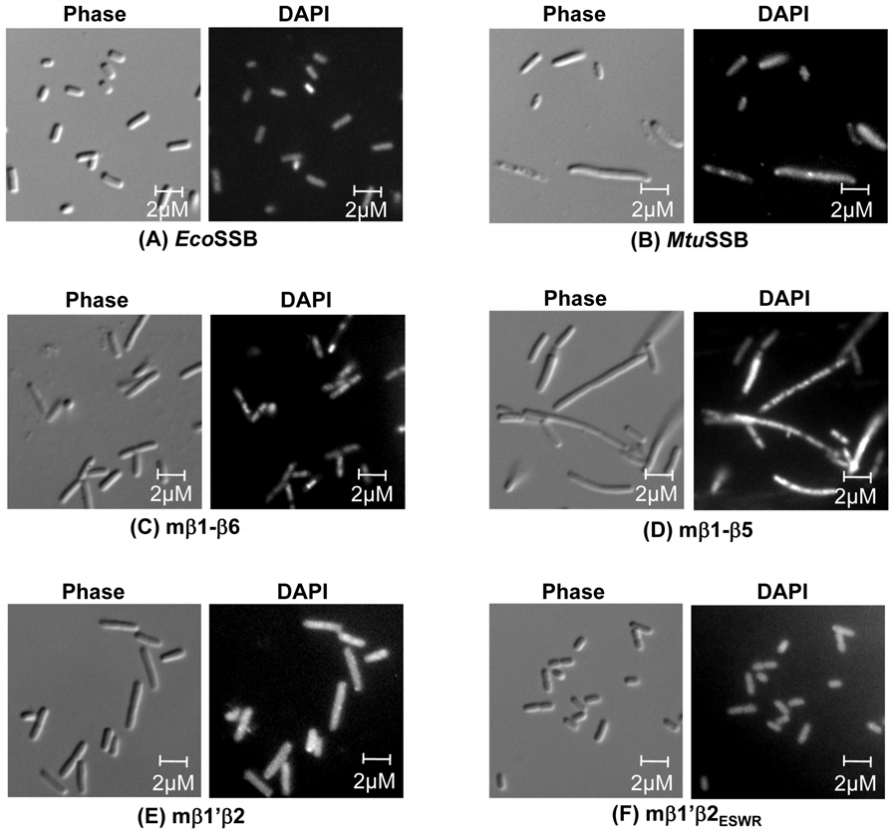
Microscopic observations of *E. coli* Δ*ssb::kan* supported by various SSB constructs. Phase contrast, and DAPI stained images, as marked, are shown on the left and right sides, respectively of each panel.

Further analysis using fluorescent microscopy revealed that the less efficiently functioning SSBs resulted in an elongated cell/filamentation phenotype of *E. coli* (Figures 8B–E). In fact, the mβ1–β5 SSB caused a notable filamentation phenotype with increased number of nucleoids per cell, as revealed by the DAPI staining (Figure 8D). Importantly, the morphology of *E. coli* cells harboring mβ1′β2_ESWR_ SSB was very similar to those harboring wild-type *Eco*SSB (Figures 8F and 8A).

## Discussion

We used the crystal structure data of *Eco*SSB [12] and *Mtu*SSB [32] to design a series of chimeric SSBs, which complement *E. coli* Δ*ssb* strain with variable efficiencies. One of the constructs, mβ1′β2_ESWR_ SSB complements the strain as well as *Eco*SSB. And, while the *Mtu*SSB, mβ1–β6 SSB and mβ1′β2 complement the strain weakly, they show a limited improvement in rescuing it in a dose dependent manner as the inducer concentration is increased (Figure 7). However, under the same conditions, the rescue offered by the mβ1–β5 SSB is poor. The mβ4–β5, mβ1 and ΔC SSBs do not rescue the strain for its growth highlighting the intricacies and significance of the specificity of inter-subunit interactions for a fully functional SSB. Furthermore, as the mβ1–β5 and mβ4–β5 SSBs are proficient in tetramerization and DNA binding, our observations suggest that the importance of inter-subunit interactions is not limited to merely provide these functions. The nature of these interactions may be crucial in allowing conformational changes (‘cross-talk’) between various regions of SSB, necessary for the *in vivo* function of SSBs. For example, in mβ1–β5 SSB, presence of *Eco*SSB sequences downstream to mβ1–β5 resulted in a change in the mode of DNA binding. However, an additional presence of *Mtu*SSB sequences (β6) in mβ1–β6 SSB resulted in a mode of binding comparable to *Eco*SSB and also resulted in better growth.

Moreover, the tip of the L_45_ loop in *Eco*SSB (Figure 1A, panel ii) undergoes a conformational change of ∼2 Å upon DNA binding [43]. The L_45_ loop at the tetramer-tetramer interface is predicted to be important for the SSB_35_ mode of DNA binding [12], [43] and is thus important in cooperativity of SSB binding to DNA. We should say that while our fluorescence reverse titrations (Table 2) do not reveal significant differences in the cooperativity of DNA binding by the SSBs, small differences, undetectable in this assay may be significant *in vivo*. The mβ4–β5 SSB possesses the L_45_ loop region from *Mtu*SSB, and while it retains the oligomeric status and DNA binding ability, it may be compromised for *in vivo* cooperativity. Recent computational analysis has indeed suggested that the movement of L_45_ loop in *Eco*SSB, *Mtu*SSB, and *Streptomyces coelicolor* SSB is different [34]. However, as the mβ1–β6 SSB construct complemented *E. coli* for the essential function of SSB, albeit less efficiently, our observations suggest that the L_45_ loop movements could be influenced by the context of the neighboring sequence. This may also be a reason why the mβ1–β5 SSB lacking the *Mtu*SSB specific region downstream of the β5 strand, is unable to offer a significant rescue of the Δ*ssb* strain of *E. coli* for its growth. Further studies would be required to understand the contributions of specific interactions of the L_45_ loop with the neighboring sequences.

Furthermore, as revealed by the native-PAGE, gel filtration chromatography and chymotrypsin digestion analyses, the mβ1 SSB wherein the β1 strand was from *Mtu*SSB, was destabilized at least in its quaternary structure and highly compromised for DNA binding, suggesting that the β1 strand is involved in specific interactions not compensated for by the heterologous sequences from *Mtu*SSB. Recently, the β1 strand of *Eco*SSB was shown to be involved in direct hydrogen bonding in monomer-monomer interactions; whereas the same region in *Mtu*SSB establishes water mediated hydrogen bonds [34]. Replacement of β1′ and β2 strands of *Eco*SSB with those from *Mtu*SSB (in mβ1′β2 SSB) also resulted in structural alterations (Figure 3). However, unlike the mβ1 SSB, the mβ1′β2 SSB is able to bind DNA and resist complete digestion by chymotrypsin (Figures 4C; 5G and 5H). Interestingly, micromanipulation of this construct by introduction of *Eco*SSB specific ‘ESWR’ sequence (in place of the *Mtu*SSB specific ‘PRIY’) important for oligomerization of SSB [34], converted the new chimera, mβ1′β2_ESWR_ SSB, into a more efficient protein (Figures 3, 5, 7 and 8).

In *Eco*SSB, W40 and W54 are important for DNA binding. In *Mtu*SSB, these residues are replaced by I39 and F54. The model of *Mtu*SSB-ssDNA reveals that the absence of W54 in *Mtu*SSB is compensated for by W60. Also, there are ten basic residues in *Mtu*SSB as opposed to six in *Eco*SSB. Additional ionic residues are predicted to compensate for the absence of W40 of *Eco*SSB. Even though the DNA binding properties of *Eco*SSB, *Mtu*SSB, and various nonfunctional chimeras are similar, the precise mode of DNA binding in them may be different due to alteration of the residues crucial in determining the mode of DNA interactions [32]. And, as is evident from the elongated cell/filamentation phenotypes of *Mtu*SSB, mβ1–β6, mβ1′β2 and mβ1–β5 SSBs, even the minor deficiencies in the varied DNA transaction activities of SSB may be significant from the *in vivo* perspective.

It had been reported that overexpression of SSB in *E. coli* results in elongated cell phenotype [44], which appeared unlikely due to a marginal overexpression (1.2 to 1.5 fold) of *sfi* gene product known to cause inhibition of cell division. In our studies, the level of expression of mβ1–β5 SSB is the same as those of *Mtu*SSB or mβ1–β6 SSB (Figure 6C). However, among these while the mβ1–β5 SSB caused a filamentation phenotype, the other two (*Mtu*SSB and mβ1–β6 SSB) resulted in a milder phenotype of elongated cells. Also, the level of expression of *Eco*SSB, and mβ1′β2_ESWR_ SSBs (readily detected by commassie blue staining of SDS-PAGE, Figure 6B) is much higher than that of *Mtu*SSB, mβ1–β6, or mβ1–β5 SSBs (immunoblotting was needed for their clear visualization, Figure 6C). However, neither the *Eco*SSB nor the mβ1′β2_ESWR_ SSB result in either an elongated cell or filamentation phenotypes. Taken together, these observations suggest that, at least in our study, the elongated cell/filamentation phenotype is not due to overexpression of SSB, but rather due to inefficient function of SSB. In fact, in a more recent report [45], it was observed that when the SSB levels were decreased, it resulted in a filamentation phenotype in *E. coli*. Importantly, further studies using mβ1–β5 SSB may prove useful in understanding the mechanism of filamentation phenotype in *E. coli*.

Finally, a recent study on *Dra*SSB having only two C-terminal tails, showed that it complemented *E. coli* for its essential function of *Eco*SSB in the ‘plasmid bumping’ assay. Hence, it was somewhat surprising that using the same assay, both in our earlier study [35] as well as the present study, we failed to see complementation of *E. coli* Δ*ssb* strain by *Mtu*SSB or some of the chimeric SSBs. Likewise, despite the fact that the DNA binding domain of *Hs*mtSSB shared similarity with the corresponding domain of *Eco*SSB, a chimera wherein the DNA binding domain of *Eco*SSB was replaced with the corresponding domain of *Hs*mtSSB [46], [47] failed to function in *E. coli* [48]. At least, in the case of *Mtu*SSB and mβ1–β6 SSB (and the other constructs), it is now clear that the conditions used for the ‘plasmid bumping’ assay did not overcome the fitness disadvantage for the *E. coli* Δ*ssb* strains to sustain exclusively on these SSBs (as opposed to those harboring both the *Eco*SSB and the test SSB). Importantly, the new assay developed in this study, overcomes the fitness disadvantage of a weakly functioning SSB by selective blocking of the replication of the parent plasmid by (*i. e.* by withdrawal of IPTG needed for the replication of pHYD*Eco*SSB). In fact, this assay allowed us to detect *in vivo* functioning of even the mβ1–β5 SSB, wherein the total viable counts, at saturation, were about three orders of magnitude lower than the strain harboring *Eco*SSB. Also, this assay has the advantage of not requiring multiple sub-culturing to bump out the original *Eco*SSB construct. Thus, we believe that the assay developed in this study may be better suited to detect activities of the SSB constructs that offer weak complementation.

## Materials and Methods

### DNA oligomers, bacterial strains and media

DNA oligomers (Table 1, S2) were obtained from Sigma-Aldrich, India. *E. coli* strains (Table 1) were grown in Luria-Bertani (LB) medium. LB-agar contained 1.6% (w/v) agar (Difco, USA). Ampicillin (Amp, 100 µg ml^−1^), kanamycin (Kan, 25 µg ml^−1^), tetracycline (Tet, 7.5 µg ml^−1^), or chloramphenicol (Cam, 15 µg ml^−1^) were added to the growth media as required.

### Cloning, overexpression and purification of SSBs and their analysis on native gels

To generate chimeric SSBs, *Eco*SSB sequences were substituted with the corresponding sequences from *Mtu*SSB (Table 1, S3, and Methods S1). SSB open reading frames were also subcloned into pET11D, pUC18R or pBAD/His B (Invitrogen) from the respective pTrc99C constructs using standard methods [49]. The pET11D based expression constructs for mβ1–β6, mβ1–β5, mβ4–β5, mβ1 and ΔC SSBs were introduced into *E coli* BL21(DE3). The pTrc99c based expression constructs for *Eco*SSB, *Mtu*SSB, mβ1′β2 SSB and mβ1′β2_ESWR_ SSB; and the pUC18R based construct for mβ1 SSB were introduced into *E. coli* TG1. Cultures (1.2 L) were grown to OD_600_ of ∼0.5 to 0.6 at 37°C under shaking, supplemented with 0.5 mM isopropyl-β-D-galactopyranoside (IPTG) and the growth continued further for 4 h. Cells were harvested and processed [31] to obtain pure SSB preparations, estimated by Bradford's method using BSA as standard, and stored in 50 mM Tris.HCl, pH 8.0, 0.1 mM Na_2_EDTA, 500 mM NaCl and 10% glycerol. Analysis of the proteins on the native polyacrylamide gels (native-PAGE) was as described before [31].

### Gel Filtration analysis of SSB proteins

Oligomeric status of various SSB proteins were determined by gel filtration chromatography. Proteins were chromatographed on Superose^™^ 6HR 10/30 column (bed volume ∼24 ml) attached to an AKTA basic FPLC (GE Healthcare Lifesciences). The column was equilibrated with buffer containing 20 mM Tris.HCl pH 8.0, 500 mM NaCl and 0.1 mM Na_2_EDTA. The flow rate was maintained at 0.3 ml^−1^ and elution profile was monitored by absorbance at 280 nm. The void volume (*Vo*) was determined by blue dextran and the column was calibrated using following standard molecular size markers: thyroglobulin (670 kDa), *Eco*SSB (76 kDa), chicken globulin (44 kDa), equine myoglobin (17 kDa), vitamin B12 (1.3 kDa). Various amounts of SSB proteins (10–200 µg) were loaded on to the column. The *Vo* of the column was found to be 7.5 ml. The elution volumes (*Ve*) of marker proteins and SSB proteins were determined and the oligomeric status of SSB proteins was determined from the plot of *Ve/Vo versus* log of molecular size markers.

### Electrophoretic mobility shift assays (EMSA)

SSBs (0.2, 2 and 10 pmol) were mixed with 5′ [^32^P] - end labeled 79mer DNA (1 pmol, ∼20,000 cpm) in 15 µl reactions containing 20 mM Tris.HCl, pH 8.0, 50 mM NaCl, 5% glycerol (v/v) and 50 µg/ml BSA, incubated for 30 min at 4°C and electrophoresed on 8% native-PAGE (30∶0.5, acrylamide∶bisacrylamide) using 1× TBE (Tris-Borate-Na_2_EDTA) for 1–2 h at 15 V cm^−1^ in cold room, and visualized by BioImage Analyzer (FLA2000, Fuji).

### Digestions of SSBs with chymotrypsin

Reactions (15 µl) with ∼2 µg of SSBs were set up [18] in buffer consisting of 10 mM Tris.HCl, pH 8.1, 0.3 M NaCl, 1 mM CaCl_2_ and 5% glycerol (v/v), incubated for 30 min on ice in the absence or presence of 2 µg sheared and heat denatured DNA. The DNA was prepared by digestion of ∼100 µg *E. coli* MG1655 genomic DNA with MspI (50 U) followed by chloroform-phenol extraction and ethanol precipitation. The digested DNA was sonicated at pulse rate of 2 s (on/off) for 1 min, heat denaturation (90°C for 30 min) and chilled on ice. The reactions were initiated by adding ∼100 ng chymotrypsin (Amresco) for various times at 37°C, stopped by adding 1× SDS sample loading dye and heating at 90°C for 5 min, and analyzed on SDS-PAGE (17.5%) followed by coomassie brilliant blue staining.

### Fluorescence titrations

Equilibrium DNA binding of SSBs was monitored by intrinsic Trp fluorescence quenching in a Fluorpmax-4 spectrofluorometer (HORIBA Jobin Yvon). SSBs (0.1 µM) were taken in 450 µl buffer A (20 mM Tris.HCl, pH 8.0, 0.1 mM Na_2_EDTA) containing 200 mM NaCl or 50 mM NaCl in 0.5 ml cuvettes. poly (dT) was added at intervals of 3–4 min and emission intensity at 354 nm (emission band pass 5 nm) was collected after excitation at 296 nm (excitation band pass 5 nm) at 25°C maintained by peltier temperature control. The poly(dT) (Sigma-Aldrich) was estimated from extinction coefficient ε_260_ = 8.1×10^3^ M^−1^ (per nucleotides) (cm^−1^) [19]–[22].

### Estimation of binding constant (*K*_obs_ mM^−1^), maximal fluorescence quenching (*Q*_max_), binding site size (*n*) and co-operativity (*ω*)

These parameters were estimated by fitting a non-linear least squares isotherm onto the data-points obtained by reverse fluorescence titration experiments. We used the binding curve fitting protocol [36] and estimated the above parameters by performing least squares minimization using the ‘Solver’ tool add-in in Microsoft Excel [37]. The equations applied to obtain initial values for *K*_obs_, *Q*_max_ and *ω* are as follows: 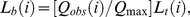
(1) where 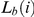 = protein bound to DNA, 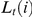 = total protein concentration, 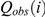 = observed fluorescence quenching, 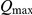 = maximal fluorescence quenching 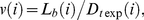
(2)where 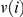 = moles of bound ligand per mole of total lattice residues, 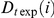 = *i* ^th^ experimental total nucleic acid concentration.

These values are substituted into the McGhee-von Hippel model (McGhee & von Hippel, 1974), as modified by Lohman and Mascotti (1992) to include both noncooperative and cooperative binding, to obtain the concentration of free protein, 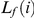: 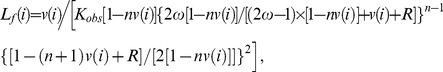
(3)

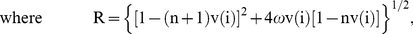
(4)

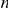
 is the site size (i.e. the number of bases occluded by binding), 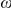 is the cooperativity parameter, and 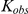 is the intrinsic binding constant observed at the specified pH and salt concentrations.

A new value for 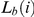 is calculated from the total protein concentration, 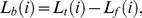
(5)and the corresponding value for the total DNA concentration, 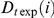, is calculated using the definition of the binding density 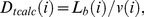
(6)

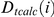
 is compared to the experimental value 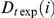, and 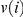 is iteratively incremented until the difference between the calculated and experimental 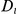 values is acceptably small (typically less than 0.01% error). The value of 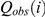 which corresponds to the final 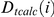 [i.e. 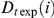], is calculated by rearrangement of eq 1: 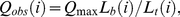
(7) Thus, 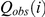 has been calculated for a given value of 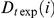.

The function requires four parameters, 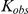, 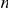, 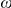, and 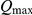, which were optimized by nonlinear regression (Bevington & Robinson, 1992).

We report the parameters that yielded the minimum value for the sum of the squared differences between the newly calculated 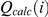 and the actual 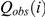.

The function requires four parameters, *K*_obs_, *n*, ω, and *Q*_max_, which were optimized by nonlinear regression [40]. The parameters reported in Table 2 yielded the minimum value for the sum of the squared differences between the newly calculated *Q*_obs_(*i*) and the actual *Q*_obs_(*i*).

### Complementation analysis

The pBAD based expression constructs were introduced into *E. coli* RDP317-1 harboring pHYD*Eco*SSB (ColE1 ori, Cam^R^) whose replication is dependent on the presence of IPTG, and the transformants selected on LB agar containing Kan, Amp and 0.02% arabinose (or Kan, Amp and 0.5 mM IPTG, as control). The isolated colonies were grown in 2 ml LB containing Kan, Amp and 0.02% arabinose to late stationary phase and streaked on LB agar containing Kan and Amp with various concentration of arabinose.

### Expression analysis of SSBs

*E. coli* TG1 strains harboring pBAD constructs of SSBs were grown to mid log phase in 2–3 ml cultures. Aliquots (1 ml) were either not supplemented or supplemented with 0.02–0.2% arabinose, and grown further for 3 h. Cells were harvested at 5000 rpm for 5 min, resuspended in 200 µl TME (25 mM Tris.HCl, pH 8.0, 2 mM β-mercaptoethanol and 1 mM Na_2_EDTA) and subjected to sonication (10 s pulses on/off; 4–5 times). The cell-free extracts were separated by centrifugation at 12000 rpm for 10 min at 4°C. Cell-free extracts (10 µg total protein) were resolved on SDS-PAGE (15%). Expression of *Eco*SSB, mβ4–β5, ΔC, mβ1, mβ1′β2, mβ1′β2_ESWR_ could be detected by coomassie blue staining. For a clear detection of *Mtu*SSB, mβ1–β6 and mβ1–β5 constructs, the resolved proteins were electroblotted onto polyvinylidene difluoride membrane (PVDF, GE Healthcare) and detected by immunoblotting [13]. Briefly, the membrane was blocked overnight with 5% non-fat dairy milk in TBST (20 mM Tris.HCl, pH7.4, 0.2% Tween 20, 150 mM NaCl), washed thrice with TBS, incubated with rabbit antisera (1∶2000 dilution) containing anti-*Mtu*SSB and anti-RRF (for loading control) polyclonal antibodies for 2 h at room temperature, washed thrice with TBS, incubated with anti rabbit goat IgG secondary antibody conjugated with HRP (horse radish peroxidase) at a dilution of 1∶2000 for 2 h, washed again with TBS, equilibrated in 10 mM Tris.HCl, pH 7.5, 150 mM NaCl and developed with 3, 3′-diaminobenzidine (DAB) in the presence of 0.03% H_2_O_2_.

### Growth curve analysis

Five independent colonies were inoculated in LB containing Kan, Amp and 0.02% arabinose to obtain late stationary phase cultures; and inoculated at 0.1% level in LB containing Kan, Amp and arabinose (as indicated) in the honeycomb microtitre plates. The growth was recorded at 600 nm using Bioscreen C growth reader (OY growth, Finland) at 37°C on hourly basis. Average values (±SEM) for three isolates were plotted.

### Microscopic studies

Fresh transformants of *E. coli* Δ*ssb* strain harboring SSB constructs (pBAD series) were grown to log phase (7–9 h in 2 ml LB containing 0.02% arabinose). Bacterial cells were collected by centrifugation at 5,000 rpm for 5 min, washed with PBS (20 mM sodium phosphate, pH 7.2 containing 0.8% NaCl), suspended in 500 µl of 4% paraformaldehyde solution in 0.1 M sodium phosphate buffer (pH 7.2), and incubated at 4°C for ∼4 h. The fixed cells were collected by centrifugation at 5000 rpm for 5 min and resuspended in 66× diluted PBS. The wells of the multi-well slide were coated with 10 µl of 0.1% (w/v) poly-L-lysine (Sigma-Aldrich) for 10 min. Poly-L-lysine was removed and 10 µl of fixed bacterial cells (appropriately diluted) were kept on the wells for 15 min, washed first with PBS and then with 66× diluted PBS. The bacterial cells were stained with 0.25 µg ml^−1^ solution of 4′, 6-diamidino-2-phenylindole (DAPI) in 66× diluted PBS for 5 min in dark, washed with PBS followed by 66× diluted PBS, and visualized in fluorescence microscope (ZEISS, Axio Imager) with 100× objective lens.

## Supporting Information

Methods S1 Generation of chimeric constructs of SSB.
(DOC)

Methods S2 Plasmid bumping experiment.
(DOC)

Figure S1 The gel filtration chromatography elution profiles of *Eco*SSB, *Mtu*SSB, mβ1–β5 SSB, mβ1–β6 SSB and ΔC SSB. Tetramer peak and *Vo* are indicated by dashed vertical lines. For further details see Figure 3.
(TIF)

Figure S2 Inverse fluorescence titrations. SSBs (0.1 µM) were titrated with increasing concentration of poly(dT) in buffer A (20 mM Tris.HCl, pH 8.0, 0.1 mM Na_2_EDTA) containing (i) 200 mM NaCl or (ii) 50 mM NaCl. The smooth curves represent the best fit data to 1∶1 model of non-linear least squares isotherm (Materials and Methods).
(TIF)

Table S1 Complementation analysis by plasmid bumping experiment.
(DOC)

Table S2 List of DNA oligomers used for generating chimeric SSBs.
(DOC)

Table S3 Nucleotide and amino acid sequences of SSB constructs.
(DOC)
