# Supplementary material for: Chimeras of Escherichia coli and Mycobacterium tuberculosis Single-Stranded DNA Binding Proteins: Characterization and Function in Escherichia coli
Source: PLoS One. 2011 Dec 12;6(12):e27216. doi: 10.1371/journal.pone.0027216 (PMC3236198; doi:10.1371/journal.pone.0027216)
Supplement: Methods S1 — Generation of chimeric constructs of SSB. (DOC) [file pone.0027216.s001.doc]

**Generation of chimeric constructs of SSB:** Standard genetic engineering methods [49] were used to generate the constructs described below. Complete amino acid sequences of the *Eco*SSB, *Mtu*SSB and the various chimeras, as deduced from the nucleotide sequence of the ORFs, are shown in Table S3.

**(A)** **pTrc mβ1-β6:** pTrc*MtuEco*SSB generated earlier [35] has been renamed as pTrc mβ1-β6.

**(B)** **pTrc mβ1-β5:** This construct was generated by introducing NheI sites in both the *Mtu*SSB and *Eco*SSB open reading frames (ORF) by mutating the DNA sequence, CTTCGG encoding _110_LR_111_ to CTAGCG encoding _110_LA_111_ in *Mtu*SSB and CTGGGT encoding _113_LG_114_ to CTAGCT encoding _113_LA_114_ in *Eco*SSB. Mutagenesis was performed by inverse PCR using *Mtu*SSB-NheI Fp, *Mtu*SSB-NheI Rp for *Mtu*SSB; and *Eco*SSB-NheI Fp, *Eco*SSB-NheI Rp primers for *Eco*SSB using Pfu DNA polymerase. PCR conditions using pTrc*Mtu*SSB template included heating it to 95 °C for 4 min followed by 25 cycles of incubations at 95 °C for 1 min, 50 °C for 45 s, and 70 °C for 11 min; and a final incubation of 10 min at 70 °C. PCR to introduce changes in *Eco*SSB ORF was done in RAMPS program wherein pTrc*Eco*SSB was heated to 94 °C for 4 min followed by 25 cycles of incubations at 94 °C for 45 s, cooling to 22 °C at the rate of 1 °C s^-1^, annealing at 22 °C for 20 s and 45 °C for 30 s, and extension at 70 °C for 14 min; and a final extension at 70 °C for 20 min. Pfu DNA polymerase (5 units or 10 units for RAMPS program) was used in a 50 μl reaction containing template (100-200 ng), primers (20 pmol each) and dNTPs 200 μM. PCR products were treated with DpnI (10 units) overnight prior to introducing into *E. coli* TG1. Transformants were screened by NheI digestions of the plasmid preparation. Subsequently, the NheI and HindIII fragment from pTrc *Eco*SSB(Nhe) was ligated into the vector backbone of NheI and HindIII digested pTrc *Mtu*SSB(Nhe) and confirmed by DNA sequencing.

**(C)** **pTrc ΔC:** During DNA sequencing, one of the clones was serendipitously identified as ΔC SSB construct. This construct contains the first 113 amino acids of *Eco*SSB, and the remaining amino acids (114 to 133) from *Mtu*SSB and vector sequence.

**(D)** **pTrc mβ4-β5:** This construct was generated from pTrc mβ1-β5 by replacing β1 to β3 and the α helix regions from *Mtu*SSB (amino acids _1_M-T_72_) with the corresponding sequence (_1_M-R_73_) from *Eco*SSB. The DNA sequence, ACCCGGGGG encoding _72_TRG_74_ and possessing an XmaI site (CCCGGG) is present in *Mtu*SSB. The *Eco*SSB-XmaI Rp was designed to mutate the CGTAAAGGT sequence of *Eco*SSB encoding _73_RKG_75_ to an XmaI site (GCCCGGGGT) encoding _73_ARG_75_. Subsequently, the N-terminal region of *Eco*SSB was amplified with *Eco*SSB- Fp and *Eco*SSB-XmaI-Rp with Pfu DNA polymerase. PCR conditions included, heating at 94 ^o^C for 4 min, followed by 24 cycles of incubations at 94 ^o^C for 1 min, 50 ^o^C for 30 s, 70 ^o^C for 30 s; and a final incubation at 70 ^o^C for 10 min. PCR product (~220 bp) was digested with NcoI and XmaI, ligated into similarly digested pTrc mβ1-β5 and confirmed by DNA sequencing.

**(E)** **pTrc mβ1:** First a pTrc*Mtu*_11_*Eco*SSB construct wherein the first 11 amino acids of *Eco*SSB were replaced with the corresponding sequence from *Mtu*SSB was generated. The DNA corresponding to the first 11 amino acids of *Mtu*SSB was amplified with pTrc Fp and M_11_*Eco*SSB-Rp Rp. PCR conditions using pTrc*Mtu*SSB as template included, heating at 94 ^o^C for 4 min followed by 24 cycles of incubations at 94 ^o^C for 1 min, 55 ^o^C for 30 s, and 70 ^o^C for 20 s. Final incubation was at 70 ^o^C for 10 min. The PCR product was purified and used as megaprimer for inverse PCR using pTrc*Eco*SSB template. For the inverse PCR, initial heating of the template at 94 ^o^C for 4 min was followed by 24 cycles of incubations at 94 ^o^C for 1 min, 52 ^o^C for 30 s, and 70 ^o^C for 11 min. Final incubation was at 70 ^o^C for 10 min. The PCR product was treated with DpnI and transformed into *E. coli* TG1. The positive clones of pTrc*Mtu*_11_*Eco*SSB were identified by the loss of MscI site. To generate pTrc mβ1, the first two amino acids (MA) of the pTrc*Mtu*_11_*Eco*SSB were replaced with the first four of *Eco*SSB (MASR) by introducing two amino acids (SR) downstream of MA. The mβ1SSB-Rp Fp and mβ1SSB-Rp Rp were used for inverse PCR using pTrc*Mtu*_11_*Eco*SSB template. To carry out PCR, reaction was heated to 94 ^o^C for 4 min followed by 24 cycles of incubations at 94 ^o^C for 1 min, 52 ^o^C for 30 s, and 70 ^o^C for 11 min. Final incubation was at 70 ^o^C for 10 min. The PCR product was treated with DpnI and introduced into *E. coli* TG1. The positive clones for were screened by the presence of MscI site and confirmed by DNA sequencing.

**(F)** **pTrc mβ1’β2 SSB:** mβ1’β2 SSB Fp and mβ1’β2 SSB Rp were designed to amplify 21-45 amino acids of *Mtu*SSB. The mβ1’β2 SSB Fp contains the nucleotide sequence (5’ GACCCCGAGCTGCGGTTC 3’) of the *Mtu*SSB ORF corresponding to amino acids _16_DPELRF_21_ and a 5’ flanking sequence (5’ GGTAATCTGGGTCAGGACCCG 3’) corresponding to amino acids _13_GNLGQDP_19_ in *Eco*SSB. The mβ1’β2 SSB Rp contains a sequence complementary to *Mtu*SSB nucleotide sequence (5’ GACCGTCAGACCGGCGAA 3’) corresponding to amino acids _41_DRQTGE_46_ and a 5’ flanking sequence complementary to *Eco*SSB sequence (5’ATGAAAGAACAG3’) encoding _47_MKEQ_50_. The two primers were used to amplify ~120 bp region from pTrc*Mtu*SSB. After initial denaturation at 94 ^o^C for 4 min, PCR was carried out by 24 cycles of incubations at 94 ^o^C for 1 min, 54 ^o^C for 30 s, 70 ^o^C for 30 s; and a final incubation at 70 ^o^C for 10 min. The PCR product was purified from 2% agarose gel and used as megaprimer for inverse PCR using pTrc*Eco*SSB template. The conditions for inverse PCR were, initial denaturation at 94 ^o^C for 4 min followed by 24 cycles of incubations at 94 ^o^C for 1 min, 50 ^o^C for 45 s, 70 ^o^C for 11 min; and a final incubation at 70 ^o^C for 10 min. Pfu DNA polymerase (5 units) was used in all PCRs, and the PCR products were treated with DpnI (5 units) overnight before introducing into *E. coli* TG1. Transformants were analyzed for the presence of *Mtu*SSB region by PCR of *ssb* ORF. As the *Mtu*SSB region in the clone brings HincII site, clones were first confirmed by digestion of the amplicon with HincII, and then by DNA sequencing.

**(G)** **pTrc mβ1’β2_ESWR_ SSB:** In the construct, mβ1’β2 SSB, the ‘PRIY’ sequence in the β2 strand of *Mtu*SSB sequence was changed to ‘ESWR’. The primers (mβ1’β2_ESWR_SSB Fp and mβ1’β2_ESWR_ SSB Rp) were used to set up an inverse PCR. After initial denaturation at 94 ^o^C for 4 min, PCR included 24 cycles of incubations at 94 ^o^C for 1 min, 55 ^o^C for 45 s, and 70 ^o^C for 11 min. Final incubation was at 70 ^o^C for 10 min. The PCR product was treated overnight with DpnI (5 units) and transformed into *E. coli* TG1. The resulting construct pTrc mβ1’β2_ESWR_ SSB was verified by DNA sequencing.

**Subcloning of various SSB chimeras into pET11D, pUC18R and pBAD/HisB:** The NcoI-HindIII fragments from pTrc mβ1-β6, pTrc mβ1-β5, pTrc ΔC, pTrc mβ4-β5 and pTrc mβ1 were ligated into similarly digested pET11D or pBAD/His B. The Eco32I-HindIII fragment from pTrc mβ1SSB was also subcloned into Ecl136II and HindIII digested pUC18R. In addition, the NcoI-HindIII fragments from pTrc*Eco*SSB, pTrc*Mtu*SSB, pTrc mβ1’β2, and pTrc mβ1’β2_ESWR_ were subcloned into similarly digested pBAD/His B.
