# Supplementary material for: Chimeras of Escherichia coli and Mycobacterium tuberculosis Single-Stranded DNA Binding Proteins: Characterization and Function in Escherichia coli
Source: PLoS One. 2011 Dec 12;6(12):e27216. doi: 10.1371/journal.pone.0027216 (PMC3236198; doi:10.1371/journal.pone.0027216)
Supplement: Methods S2 — Plasmid bumping experiment. (DOC) [file pone.0027216.s002.doc]

**Complementation analysis by plasmid bumping:** The complementation analysis by ‘plasmid bumping’ was carried out using *E. coli* RDP317 strain (Δ*ssb*::*kan*) harboring a wild-type *ssb* gene on plasmid pRPZ150 (ColE1 ori Tet^R^) [41,42]. The pTrc99c based test *ssb* constructs (ColE1 ori, Amp^R^) were introduced into this strain and the transformants were grown in 20 ml LB containing Amp, Kan and 0.5 mM IPTG. After 4 consecutive sub-culturing in the same media, the cultures were streaked on to LB-agar containing Amp. The isolated colonies were then patched on plates containing single antibiotic (Tet) and double antibiotic (Kan and Amp).
