# Supplementary figures and images for: Chimeras of Escherichia coli and Mycobacterium tuberculosis Single-Stranded DNA Binding Proteins: Characterization and Function in Escherichia coli
Source: PLoS One. 2011 Dec 12;6(12):e27216. doi: 10.1371/journal.pone.0027216 (PMC3236198; doi:10.1371/journal.pone.0027216)

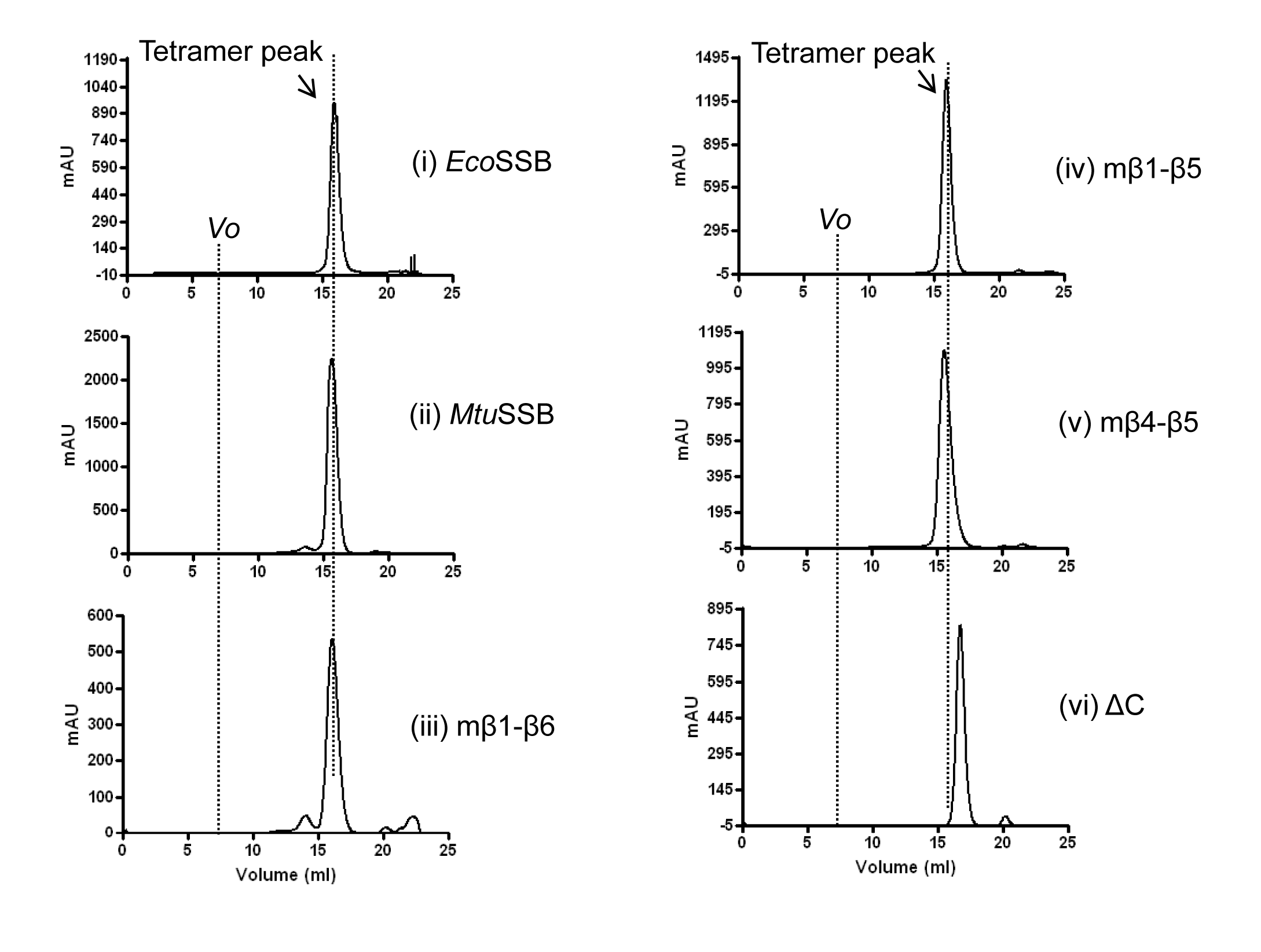

Supplement: Figure S1 — The gel filtration chromatography elution profiles of EcoSSB, MtuSSB, mβ1–β5 SSB, mβ1–β6 SSB and ΔC SSB. Tetramer peak and Vo are indicated by dashed vertical lines. For further details see Figure 3. (TIF) [file pone.0027216.s003.tif]

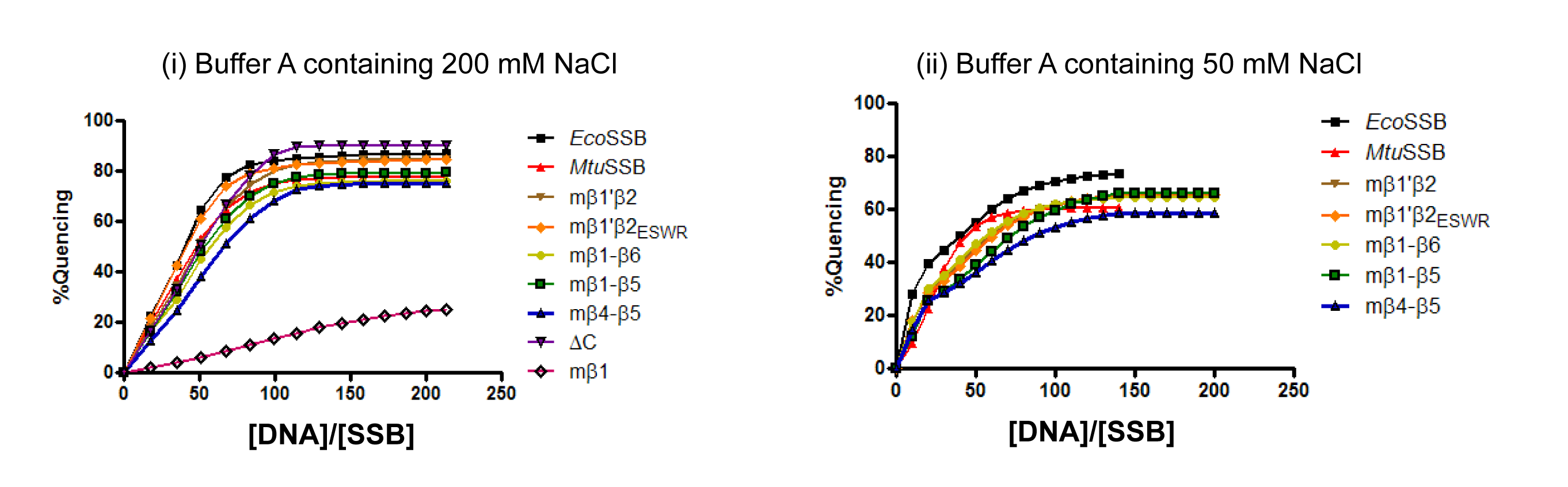

Supplement: Figure S2 — Inverse fluorescence titrations. SSBs (0.1 µM) were titrated with increasing concentration of poly(dT) in buffer A (20 mM Tris.HCl, pH 8.0, 0.1 mM Na2EDTA) containing (i) 200 mM NaCl or (ii) 50 mM NaCl. The smooth curves represent the best fit data to 1∶1 model of non-linear least squares isotherm (Materials and Methods). (TIF) [file pone.0027216.s004.tif]
