# Supplementary material for: Chimeras of Escherichia coli and Mycobacterium tuberculosis Single-Stranded DNA Binding Proteins: Characterization and Function in Escherichia coli
Source: PLoS One. 2011 Dec 12;6(12):e27216. doi: 10.1371/journal.pone.0027216 (PMC3236198; doi:10.1371/journal.pone.0027216)
Supplement: Table S1 — Complementation analysis by plasmid bumping experiment. (DOC) [file pone.0027216.s005.doc]

**Table S1: Complementation analysis by plasmid bumping experiment**

| Tester *ssb* constructs | No. of Colonies (Amp^R^, Kan^R^) | No. of Colonies (Tc^R^) | Efficiency of plasmid replacement (Tc^R^ to Tc^S^) in the Δ*ssb* strain (%) |
| --- | --- | --- | --- |
| pTrc *Eco*SSB | 30 | 0 | 100 |
| pTrc *Mtu*SSB | 30 | 30 | 0 |
| pTrc mβ1-β6 SSB | 30 | 30 | 0 |
| pTrc mβ1-β5 SSB | 30 | 30 | 0 |
| pTrc ΔSSB | 30 | 30 | 0 |
| pTrc mβ4-β5 SSB | 30 | 30 | 0 |
| pTrc mβ1 SSB | 30 | 30 | 0 |
| pTrc mβ1’β2 SSB | 30 | 30 | 0 |
| pTrc mβ1’β2_ESWR_ SSB | 30 | 0 | 100 |
