# Supplementary material for: Chimeras of Escherichia coli and Mycobacterium tuberculosis Single-Stranded DNA Binding Proteins: Characterization and Function in Escherichia coli
Source: PLoS One. 2011 Dec 12;6(12):e27216. doi: 10.1371/journal.pone.0027216 (PMC3236198; doi:10.1371/journal.pone.0027216)
Supplement: Table S2 — List of DNA oligomers used for generating chimeric SSBs. (DOC) [file pone.0027216.s006.doc]

**Table S2: List of DNA oligomers used for generating chimeric SSBs**

| **Primers** | **Sequence (5’-3’)** |
| --- | --- |
| *Eco*SSB-NheI Fp | CATGCAGATGCTAGCTGGTCGTCAGGG |
| *Eco*SSB-NheI Rp | CCCTGACGACCAGCTAGCATCTGCATG |
| *Eco*SSB Fp | GGAATTCACCATGGCCAGCAGAGG |
| *Eco*SSB-XmaI Rp | AACCTGAGAACCCCGGGCCAGATATTCGCT |
| *Mtu*SSB-NheI FP | TTGGG CC TTCGCTAGCGTACGCCACCGC |
| *Mtu*SSB-NheI Rp | GCGGTGGCGTACGCTAGCGAAGGCCCAA |
| pTrc-Bcl Rp | GGCTGTTTTGGCGGATGAGAGA |
| pTrc Fp | TAACAAGCTTACACAGGAAACAG |
| M_11_*Eco*SSB-Rp Rp | CGGGTCCTGACCCAGATTTCCGACGATG |
| mβ1SSB Fp | GAAACAGACCATGGCCAGCAGAGGTGACACCACCATC |
| mβ1SSB Rp | GATGGTGGTGTCACCTCTGCTGGCCATGGTCTGTTTC |
| mβ1’β2SSB Fp | GGTAATCTGGGTCAGGACCCCGAGCTGCGGTTC |
| mβ1’β2SSB Rp | CTGTTCTTTCATTTCGCCGGTCTGACGGTC |
| mβ1’β2_ESWR_SSB Fp | CGTGGCGTCAACGGAATCCTGGCGTGACCGTCAGACC |
| mβ1’β2_ESWR_SSB Rp | GGTCTGACGGTCACGCCAGGATTCCGTTGACGCCACG |
