# Supplementary material for: Chimeras of Escherichia coli and Mycobacterium tuberculosis Single-Stranded DNA Binding Proteins: Characterization and Function in Escherichia coli
Source: PLoS One. 2011 Dec 12;6(12):e27216. doi: 10.1371/journal.pone.0027216 (PMC3236198; doi:10.1371/journal.pone.0027216)
Supplement: Table S3 — Nucleotide and amino acid sequences of SSB constructs. (DOC) [file pone.0027216.s007.doc]

**Table S3: Nucleotide and amino acid sequences of SSB constructs**

| **SSB constructs** |
| --- |
| ***Eco*SSB** |
| **atggccagcagaggcgtaaacaaggttattctcgttggtaatctgggtcaggacccggaa**  **M A S R G V N K V I L V G N L G Q D P E 20**  **gtacgctacatgccaaatggtggcgcagttgccaacattacgctggctacttccgaatcc**  **V R Y M P N G G A V A N I T L A T S E S 40**  **tggcgtgataaagcgaccggcgagatgaaagaacagactgaatggcaccgcgttgtgctg**  **W R D K A T G E M K E Q T E W H R V V L 60**  **ttcggcaaactggcagaagtggcgagcgaatatctgcgtaaaggttctcaggtttatatc**  **F G K L A E V A S E Y L R K G S Q V Y I 80**  **gaaggtcagctgcgtacccgtaaatggaccgatcaatccggtcaggatcgctacaccaca**  **E G Q L R T R K W T D Q S G Q D R Y T T 100**  **gaagtcgtggtgaacgttggcggcaccatgcagatgctgggtggtcgtcagggtggtggc**  **E V V V N V G G T M Q M L G G R Q G G G 120**  **gctccggcaggtggcaatatcggtggtggtcagccgcagggcggttggggtcagcctcag**  **A P A G G N I G G G Q P Q G G W G Q P Q 140**  **cagccgcagggtggcaatcagttcagcggcggcgcgcagtctcgcccgcagcagtccgct**  **Q P Q G G N Q F S G G A Q S R P Q Q S A 160**  **ccggcagcgccgtctaacgagccgccgatggactttgatgatgacattccgttctga**  **P A A P S N E P P M D F D D D I P F - 178** |
| ***Mtu*SSB** |
| **atggctggtgacaccaccatcaccatcgtcggaaatctgaccgctgaccccgagctgcgg**  **M A G D T T I T I V G N L T A D P E L R 20**  **ttcaccccgtccggtgcggccgtggcgaatttcaccgtggcgtcaacgccccggatctat**  **F T P S G A A V A N F T V A S T P R I Y 40**  **gaccgtcagaccggcgaatggaaagacggcgaagcgctgttcctccggtgcaatatctgg**  **D R Q T G E W K D G E A L F L R C N I W 60**  **cgggaggcggccgagaacgtggccgagagcctcacccggggggcacgagtcatcgttagc**  **R E A A E N V A E S L T R G A R V I V S 80**  **gggcggcttaagcagcggtcgtttgaaacccgtgagggcgagaagcgcaccgtcatcgag**  **G R L K Q R S F E T R E G E K R T V I E 100**  **gtcgaggtcgatgagattgggccttcgcttcggtacgccaccgccaaggtcaacaaggcc**  **V E V D E I G P S L R Y A T A K V N K A 120**  **agccgcagcggcgggtttggcagcggatcccgtccggcgccggcgcagaccagcagcgcc**  **S R S G G F G S G S R P A P A Q T S S A 140**  **tcgggagatgacccgtggggcagcgcaccggcgtcgggttcgttcggcggcggcgatgac**  **S G D D P W G S A P A S G S F G G G D D 160**  **gaaccgccattctga**  **E P P F - 164** |
| **mβ1-β6 SSB** |
| **atggctggtgacaccaccatcaccatcgtcggaaatctgaccgctgaccccgagctgcgg**  **M A G D T T I T I V G N L T A D P E L R 20**  **ttcaccccgtccggtgcggccgtggcgaatttcaccgtggcgtcaacgccccggatctat**  **F T P S G A A V A N F T V A S T P R I Y 40**  **gaccgtcagaccggcgaatggaaagacggcgaagcgctgttcctccggtgcaatatctgg**  **D R Q T G E W K D G E A L F L R C N I W 60**  **cgggaggcggccgagaacgtggccgagagcctcacccggggggcacgagtcatcgttagc**  **R E A A E N V A E S L T R G A R V I V S 80**  **gggcggcttaagcagcggtcgtttgaaacccgtgagggcgagaagcgcaccgtcatcgag**  **G R L K Q R S F E T R E G E K R T V I E 100**  **gtcgaggtcgatgagattgggccttcgctagcgtacgccaccgccaaggtcaacaaggcc**  **V E V D E I G P S L A Y A T A K V N K A 120**  **agccgcagcggcgggtttggcagcggatcccagccgcagggcggttggggtcagcctcag**  **S R S G G F G S G S Q P Q G G W G Q P Q 140**  **cagccgcagggtggcaatcagttcagcggcggcgcgcagtctcgcccgcagcagtccgct**  **Q P Q G G N Q F S G G A Q S R P Q Q S A 160**  **ccggcagcgccgtctaacgagccgccgatggactttgatgatgacattccgttctga**  **P A A P S N E P P M D F D D D I P F - 178** |
| **mβ1-β5 SSB** |
| **atggctggtgacaccaccatcaccatcgtcggaaatctgaccgctgaccccgagctgcgg**  **M A G D T T I T I V G N L T A D P E L R 20**  **ttcaccccgtccggtgcggccgtggcgaatttcaccgtggcgtcaacgccccggatctat**  **F T P S G A A V A N F T V A S T P R I Y 40**  **gaccgtcagaccggcgaatggaaagacggcgaagcgctgttcctccggtgcaatatctgg**  **D R Q T G E W K D G E A L F L R C N I W 60**  **cgggaggcggccgagaacgtggccgagagcctcacccggggggcacgagtcatcgttagc**  **R E A A E N V A E S L T R G A R V I V S 80**  **gggcggcttaagcagcggtcgtttgaaacccgtgagggcgagaagcgcaccgtcatcgag**  **G R L K Q R S F E T R E G E K R T V I E 100**  **gtcgaggtcgatgagattgggccttcgctagcgggtggtcgtcagggtggtggcgctccg**  **V E V D E I G P S L A G G R Q G G G A P 120**  **gcaggtggcaatatcggtggtggtcagccgcagggcggttggggtcagcctcagcagccg**  **A G G N I G G G Q P Q G G W G Q P Q Q P 140**  **cagggtggcaatcagttcagcggcggcgcgcagtctcgcccgcagcagtccgctccggca**  **Q G G N Q F S G G A Q S R P Q Q S A P A 160**  **gcgccgtctaacgagccgccgatggactttgatgatgacattccgttctga**  **A P S N E P P M D F D D D I P F - 176** |
| **mβ4-β5 SSB** |
| **atggccagcagaggcgtaaacaaggttattctcgttggtaatctgggtcaggacccggaa**  **M A S R G V N K V I L V G N L G Q D P E 20**  **gtacgctacatgccaaatggtggcgcagttgccaacattacgctggctacttccgaatcc**  **V R Y M P N G G A V A N I T L A T S E S 40**  **tggcgtgataaagcgaccggcgagatgaaagaacagactgaatggcaccgcgttgtgctg**  **W R D K A T G E M K E Q T E W H R V V L 60**  **ttcggcaaactggcagaagtggcgagcgaatatctggcccggggggcacgagtcatcgtt**  **F G K L A E V A S E Y L A R G A R V I V 80**  **agcgggcggcttaagcagcggtcgtttgaaacccgtgagggcgagaagcgcaccgtcatc**  **S G R L K Q R S F E T R E G E K R T V I 100**  **gaggtcgaggtcgatgagattgggccttcgctagctggtcgtcagggtggtggcgctccg**  **E V E V D E I G P S L A G R Q G G G A P 120**  **gcaggtggcaatatcggtggtggtcagccgcagggcggttggggtcagcctcagcagccg**  **A G G N I G G G Q P Q G G W G Q P Q Q P 140**  **cagggtggcaatcagttcagcggcggcgcgcagtctcgcccgcagcagtccgctccggca**  **Q G G N Q F S G G A Q S R P Q Q S A P A 160**  **gcgccgtctaacgagccgccgatggactttgatgatgacattccgttctga**  **A P S N E P P M D F D D D I P F - 176** |
| **mβ1 SSB** |
| **atggccagcagaggtgacaccaccatcaccatcgtcggtaatctgggtcaggacccggaa**  **M A S R G D T T I T I V G N L G Q D P E 20**  **gtacgctacatgccaaatggtggcgcagttgccaacattacgctggctacttccgaatcc**  **V R Y M P N G G A V A N I T L A T S E S 40**  **tggcgtgataaagcgaccggcgagatgaaagaacagactgaatggcaccgcgttgtgctg**  **W R D K A T G E M K E Q T E W H R V V L 60**  **ttcggcaaactggcagaagtggcgagcgaatatctgcgtaaaggttctcaggtttatatc**  **F G K L A E V A S E Y L R K G S Q V Y I 80**  **gaaggtcagctgcgtacccgtaaatggaccgatcaatccggtcaggatcgctacaccaca**  **E G Q L R T R K W T D Q S G Q D R Y T T 100**  **gaagtcgtggtgaacgttggcggcaccatgcagatgctgggtggtcgtcagggtggtggc**  **E V V V N V G G T M Q M L G G R Q G G G 120**  **gctccggcaggtggcaatatcggtggtggtcagccgcagggcggttggggtcagcctcag**  **A P A G G N I G G G Q P Q G G W G Q P Q 140**  **cagccgcagggtggcaatcagttcagcggcggcgcgcagtctcgcccgcagcagtccgct**  **Q P Q G G N Q F S G G A Q S R P Q Q S A 160**  **ccggcagcgccgtctaacgagccgccgatggactttgatgatgacattccgttctga**  **P A A P S N E P P M D F D D D I P F - 178** |
| **mβ1’β2SSB** |
| **atggccagcagaggcgtaaacaaggttattctcgttggtaatctgggtcaggaccccgag**  **M A S R G V N K V I L V G N L G Q D P E 20**  **ctgcggttcaccccgtccggtgcggccgtggcgaatttcaccgtggcgtcaacgccccgg**  **L R F T P S G A A V A N F T V A S T P R 40**  **atctatgaccgtcagaccggcgaaatgaaagaacagactgaatggcaccgcgttgtgctg**  **I Y D R Q T G E M K E Q T E W H R V V L 60**  **ttcggcaaactggcagaagtggcgagcgaatatctgcgtaaaggttctcaggtttatatc**  **F G K L A E V A S E Y L R K G S Q V Y I 80**  **gaaggtcagctgcgtacccgtaaatggaccgatcaatccggtcaggatcgctacaccaca**  **E G Q L R T R K W T D Q S G Q D R Y T T 100**  **gaagtcgtggtgaacgttggcggcaccatgcagatgctgggtggtcgtcagggtggtggc**  **E V V V N V G G T M Q M L G G R Q G G G 120**  **gctccggcaggtggcaatatcggtggtggtcagccgcagggcggttggggtcagcctcag**  **A P A G G N I G G G Q P Q G G W G Q P Q 140**  **cagccgcagggtggcaatcagttcagcggcggcgcgcagtctcgcccgcagcagtccgct**  **Q P Q G G N Q F S G G A Q S R P Q Q S A 160**  **ccggcagcgccgtctaacgagccgccgatggactttgatgatgacattccgttctga**  **P A A P S N E P P M D F D D D I P F - 178** |
| **mβ1’β2_ESWR_SSB** |
| **atggccagcagaggcgtaaacaaggttattctcgttggtaatctgggtcaggaccccgag**  **M A S R G V N K V I L V G N L G Q D P E 20**  **ctgcggttcaccccgtccggtgcggccgtggcgaatttcaccgtggcgtcaacggaatcc**  **L R F T P S G A A V A N F T V A S T E S 40**  **tggcgtgaccgtcagaccggcgaaatgaaagaacagactgaatggcaccgcgttgtgctg**  **W R D R Q T G E M K E Q T E W H R V V L 60**  **ttcggcaaactggcagaagtggcgagcgaatatctgcgtaaaggttctcaggtttatatc**  **F G K L A E V A S E Y L R K G S Q V Y I 80**  **gaaggtcagctgcgtacccgtaaatggaccgatcaatccggtcaggatcgctacaccaca**  **E G Q L R T R K W T D Q S G Q D R Y T T 100**  **gaagtcgtggtgaacgttggcggcaccatgcagatgctgggtggtcgtcagggtggtggc**  **E V V V N V G G T M Q M L G G R Q G G G 120**  **gctccggcaggtggcaatatcggtggtggtcagccgcagggcggttggggtcagcctcag**  **A P A G G N I G G G Q P Q G G W G Q P Q 140**  **cagccgcagggtggcaatcagttcagcggcggcgcgcagtctcgcccgcagcagtccgct**  **Q P Q G G N Q F S G G A Q S R P Q Q S A 160**  **ccggcagcgccgtctaacgagccgccgatggactttgatgatgacattccgttctga**  **P A A P S N E P P M D F D D D I P F - 178** |
| **ΔC SSB** |
| **atggccagcagaggcgtaaacaaggttattctcgttggtaatctgggtcaggacccggaa**  **M A S R G V N K V I L V G N L G Q D P E 20**  **gtacgctacatgccaaatggtggcgcagttgccaacattacgctggctacttccgaatcc**  **V R Y M P N G G A V A N I T L A T S E S 40**  **tggcgtgataaagcgaccggcgagatgaaagaacagactgaatggcaccgcgttgtgctg**  **W R D K A T G E M K E Q T E W H R V V L 60**  **ttcggcaaactggcagaagtggcgagcgaatatctgcgtaaaggttctcaggtttatatc**  **F G K L A E V A S E Y L R K G S Q V Y I 80**  **gaaggtcagctgcgtacccgtaaatggaccgatcaatccggtcaggatcgctacaccaca**  **E G Q L R T R K W T D Q S G Q D R Y T T 100**  **gaagtcgtggtgaacgttggcggcaccatgcagatgctagcggccgagctcaacaaggcc**  **E V V V N V G G T M Q M L A A E L N K A 120**  **cgaaaggaagctgagttggctgctgccaccgctgagcaataa**  **R K E A E L A A A T A E Q - 133** |

**Note:** Sequences shown in green and red are of EcoSSB and MtuSSB origin, respectively. Sequences shown in blue are mutations incorporated to create a desired restriction site to generate the chimeric construct. Sequences shown in black in the ΔC SSB construct are of vector origin. Numbers shown on the right hand side denote the amino acid residue numbers.
